# A Review of Qualitative Research of Perception and Experiences of Dementia Among Adults From Black, African, and Caribbean Background: What and Whom Are We Researching?

**DOI:** 10.1093/geront/gnaa004

**Published:** 2021-07-13

**Authors:** Moïse Roche, Paul Higgs, Jesutofunmi Aworinde, Claudia Cooper

**Affiliations:** 1 Division of Psychiatry, University College London, UK; 2 Cicely Saunders Institute of Palliative Care, Policy & Rehabilitation, King’s College London, UK

**Keywords:** Ethnic discordance, Cultural nuance, Diversity and identity, Racial terminology issues

## Abstract

**Background and Objectives:**

Black, African, and Caribbean (BAC) families are disproportionately affected by dementia but engage less with services. Studies reporting their experiences of dementia have tended to aggregate people from diverse backgrounds, without considering the impact of this diversity, or researchers’ ethnicities. We investigated participants’ and researchers’ ethnic identities, exploring how this relates to findings.

**Research Design and Methods:**

We searched electronic databases in September 2018, for qualitative studies exploring how participants of Black ethnicity understand and experience dementia and dementia care. We reported participants’ and researchers’ ethnicities, and meta-synthesized qualitative findings regarding how ethnicity influences experiences and understanding of dementia.

**Results:**

Twenty-eight papers reported 25 studies; in United States (*n* = 17), United Kingdom (*n* = 7), and Netherlands (*n* = 1). 350/492 (71%) of participants were in U.S. studies and described as African American; participants in U.K. studies as Caribbean (*n* = 45), African/Caribbean (*n* = 44), African (*n* = 28), Black British (*n* = 7), or Indo-Caribbean (*n* = 1); and in Netherlands as Surinamese Creole (*n* = 17). 6/25 (24%) of studies reported involving recruiters/interviewers matching participants’ ethnicity; and 14/25 (56%) involved an author/advisor from a BAC background during analysis/procedures. We identified four themes: Dementia does not relate to me; Inappropriate and disrespectful services; Kinship and responsibility; Importance of religion.

**Discussion and Implications:**

Studies were mostly from a U.S. African American perspective, by researchers who were not of BAC background. Themes of dementia diagnosis and services feeling less relevant to participants than the majority population resonated across studies. We caution against the racialization of these findings, which can apply to many differing minority groups.

Addressing inequalities in access to dementia health and care services is a priority in research and practice and for racially and ethnically diverse families facing cognitive loss. A growing body of research has sought to do this, but classification systems in existing studies often inappropriately homogenize populations (Agyemang, Bhopal, & Bruijnzeels, 2005). Terms used to describe different ethnic and cultural groups vary across Europe, North America, and Australasia, where the majority of this research has been conducted. There is a danger that this translates to the body of research that uses these terms interchangeably. Qualitative dementia research is not excluded from these difficulties, albeit its primary purpose is to provide “thick description” that foregrounds nuance and cultural distinctiveness (Geertz, 1973). This paper systematically reviews qualitative work exploring the experiences of dementia of people these studies have variously described as belonging to a Black, African, and Caribbean (BAC) ethnic group. It argues that not only are different terms and categories used to describe populations on different continents but that little cultural nuance is brought out of research, often conducted by people with limited direct experience of these categories.

Henceforth, we use the term “BAC” or where we are reflecting, the terminology used in the literature being discussed. We acknowledge the ethical complexities and lack of consensus on preferred terminology, yet recognize the clear need to write about, and research this important topic in inequality.

## Background

In Europe and North America, the number and age of BAC populations are rising rapidly (He, Goodkind, & Kowal, 2016; Ortman, Velkoff, & Hogan, 2014), as is the number of older people with dementia within these populations. In 2011, a report estimated that there were 25,000 Black and Minority Ethnic (BME) people living with dementia in the United Kingdom, with a projected seven-fold increase to 172,000 by 2051 (Knapp et al., 2007). Dementia age-standardized rates are observed to be consistently higher in BAC ethnic groups than in other minority populations (Mehta & Yeo, 2017) and the majority population (Adelman, Blanchard, Rait, Leavey, & Livingston, 2011; Mayeda, Glymour, Quesenberry, & Whitmer, 2016). Despite this increased risk, U.K. Black older adults are less likely to receive a timely diagnosis or support and treatment for dementia (Adelman et al., 2011; Pham et al., 2018; Tuerk & Sauer, 2015). This apparent inequality in dementia service provision has led to a burgeoning of research into how BAC families understand dementia and experience dementia care.

For studies to inform our understanding of how different people experience dementia, we must be able to understand the ethnicity and cultural backgrounds of study populations (Ford & Kelly, 2005). Health and social researchers routinely use predefined administrative labels that vary across countries, often without a shared understanding among research communities of the taxonomy used (McKenzie & Crowcroft, 1996). While the term “White” and its synonym “Caucasian” have largely bypassed the quest for appropriate nomenclature and terminology, the term “Black” and its derivatives (e.g., BME, African American, etc.) (Bhrolcháin, 1990; Botsford, Clarke, & Gibb, 2011) have altered across time and place, mainly for political reasons (McKenzie & Crowcroft, 1994). Some scientists have argued for phasing out “the color term Black” for concealing heterogeneity and being racially charged in favor of more meaningful and detailed categories (Agyemang et al., 2005; Aspinall, 2011). For example, by using African as a prefix for other ethnic labels such as African Caribbean or African American. However, because many people who are presumed to share ethnic background do not identify as African or share common heritage, the issue of concealment of heterogeneity of some groups remains. This also introduces the issue of aggregating racially and ethnically differing people who identify as African. Population studies have found no clear preference or consensus for either “Black or African American” among the people directly affected by this terminology. There is however a seemingly universal recognition in the group representation of the term “Black,” and a global acceptance among populations as it is increasingly used in social trends and powerful campaigns such as “Black History Month” and “Black Lives Matter.” Given that people are diverse, and ethnicity is dynamic, often self-classified, sometimes presumed, and a source of continuing debate, it has become increasingly challenging to interpret the results of studies that use these labels to homogenize disparate groups of people into collectivities (Aspinall, 2002; Bhopal & Donaldson, 1998; Cole, 1993; Polenberg, 1980). Adding to the challenge are globalization and migration, eroding the boundaries that once “segmented” group culture. This review reflects on these challenges to synthesize the current literature regarding what is known about the people that studies have described as “Black” and those researching them, and how these participants have perceived and experienced dementia and its care options and whether there are “cultural” nuances that explain their interaction with this illness. Culture is a challenging and dynamic concept, which varies largely between societies and within social groups. It does not necessarily apply to everyone in a particular culture (Corin, 1995). It is also a dimension of ethnicity that is related to ideas of race in that they denote membership of a social group in which member shares presumed and/or apparent similarities that distinguish them from other groups (Bradby, 1995; Zenner, 1996). In this review, we used the idea of cultural nuance to denote what might have distinguished the population under review from other groups within specific societies. Our research questions were:

1. “How do BAC adults with dementia, their families, and other stakeholders perceive, understand, and experience dementia and dementia care services?”
2. “What do we know about the ethnic and cultural identity of the participants and researchers in the included studies?”

## Methods

We conducted a review of qualitative literature using systematic procedures to answer our first research question mentioned above.

To address our second research question, we elicited from the included papers any ethnic and cultural description of (i) participants, (ii) researchers, and (iii) explicit report of including the perspective of a person with lived experience of the ethnic and culture being examined in analyses; and (iv) author contact details.

### Search Strategy

The study main search strategy was concluded in September 2018 and updated in October 2019 by using the following electronic databases: PsychINFO, EMBASE, Medline/PubMed, Social Care Online, Web of Science, CINAHL, Cochrane, OpenGrey, Ethos, and Google Scholar. We developed our search terms in consultation with stakeholders who were carers of people with dementia and self-identified as Black British and Caribbean (*n* = 2) and Alzheimer’s Society representatives (*n* = 3). They were: “dementia,” “Alzheimer,” “Black,” “BME,” “BAME,” “ethnic,” “Culturally Diverse Communities,” “CALD” (Culturally and Linguistically Diverse). Each of these key words and phrases was used singly and in combination using the Boolean operator “AND” and “OR.” Our initial search terms included “African” and “Caribbean” which were removed as they did not yield any additional relevant studies. We augmented our electronic search with backward and forward reference searching of included articles and carried out manual searches in books (Khan et al., 2015; Torres, 2019). Once we concluded searching for all potential articles, a database alert system was designed to capture new publications. Four eligible studies were identified from the database alerts and all searches were concluded in October 2019.

### Study Selection

#### Inclusion criteria

We included studies that responded to our research question and distinguished the views and experiences of dementia of BAC participants from those of other ethnic groups. We only included papers reporting qualitative research as this was the methodology that was appropriate to our research question. No restrictions were applied for dates of publication and locations. We only included studies in English and French. We excluded reviews, conference proceedings, and media articles.

#### Screening and data extraction

M. Roche and J. Aworinde screened titles and abstracts separately, in accordance with the inclusion criteria. Any discrepancies were resolved through discussion with the other authors. All papers judged relevant to the aims of the review were read in full and data extracted as shown in Table 2.

#### Quality assessment

We assessed the quality of included studies using CASP Qualitative Checklist Section A (CASP, 2018), which comprises six items, listed below:

1. Was there a clear statement of the aims of the research?
2. Is a qualitative methodology appropriate?
3. Was the research design appropriate to address the aims of the research?
4. Was the recruitment strategy appropriate to the aims of the research?
5. Were the data collected in a way that addressed the research issue?
6. Has the relationship between researcher and participants been adequately considered?

We assigned a maximum of one point per item, with possible scores ranging from 0 (lowest quality) to 6 (highest quality) (see Table 1). We did not exclude low-quality studies as there is no consensus and limited evidence on how to approach exclusion of qualitative studies from reviews on the basis of quality (Campbell et al., 2012; Lawrence, Fossey, Ballard, Moniz-Cook, & Murray, 2012; Thomas & Harden, 2008).

### Analysis

We were guided by Thomas and Harden (2008) recommendations on synthesizing qualitative research. Using thematic analysis, we analyzed key data from the result sections of included papers. By key data, we intended to include direct quotations from BAC participants, which we prioritized over researchers’ interpretations. M. Roche and J. Aworinde independently free-coded the extracted data line by line according to the review question using NVivo12. To begin, we coded data for each country separately to check for patterns of differences, which we did not find. We developed a coding framework. We looked for similarities and differences between codes to group them into themes, while creating new codes to capture the meanings of groups of initial codes. Finally, through iterative and reflexive processes of comparisons and interpretations, we inferred higher order meanings from our themes, which we captured in more abstract and analytical concepts about how people of BAC ethnicity understand and experience dementia and its care options.

We included in our analysis data from all participants described as Black, African, and Caribbean, combined with American, British, and French.

To determine extent to which studies were informed by BAC people, we first reviewed whether studies reported involving recruiters/interviewers and authors whose ethnicities matched those of participants. Where this information was not available, we contacted corresponding authors to ask which members of the authorship team self-identified as being of BAC background. For papers we received no response, we searched Google, ResearchGate, and universities’ websites to which authors were affiliated to verify whether this information was available or determinable from their profiles. Where researchers’ biography did not specify their ethnicity, we made a determination based on appearance (skin color, facial features, hair texture). We reported this, after having considered carefully the ethics of making this judgment, given that presumed and/or assigned ethnicity, albeit widely used, can differ to self-identified ethnicity (Torres, 2019). We appreciate that this approach can be seen as problematic. The definitions of race, ethnicity, and culture are all contingent, combining both subjectivist and objectivist approaches (Brubaker, 2016); however, identifications of race and ethnicity also draw on commonplace assumptions which are readily socially understandable and are used in the framing of research questions in this area of research. We have used this later “general sense” of race and ethnicity, not as a scientific category but rather as a way of further interrogating our research data. Additionally, the authors of BAC ethnicity of this paper considered that on balance this methodology was preferable to drawing a likely erroneous conclusion that a person with lived experience of BAC ethnicity was not involved in analysis of a paper, if in publicly available data their appearance was clearly consistent with having this experience.

Our review team consisted of four researchers of different backgrounds. M. Roche self-identifies as French Black Caribbean; P. Higgs as White Irish; J. Aworinde as Black British of African descent; and C. Cooper as White British.

## Results

### Study Characteristics and Quality Appraisal

**Figure 1:**
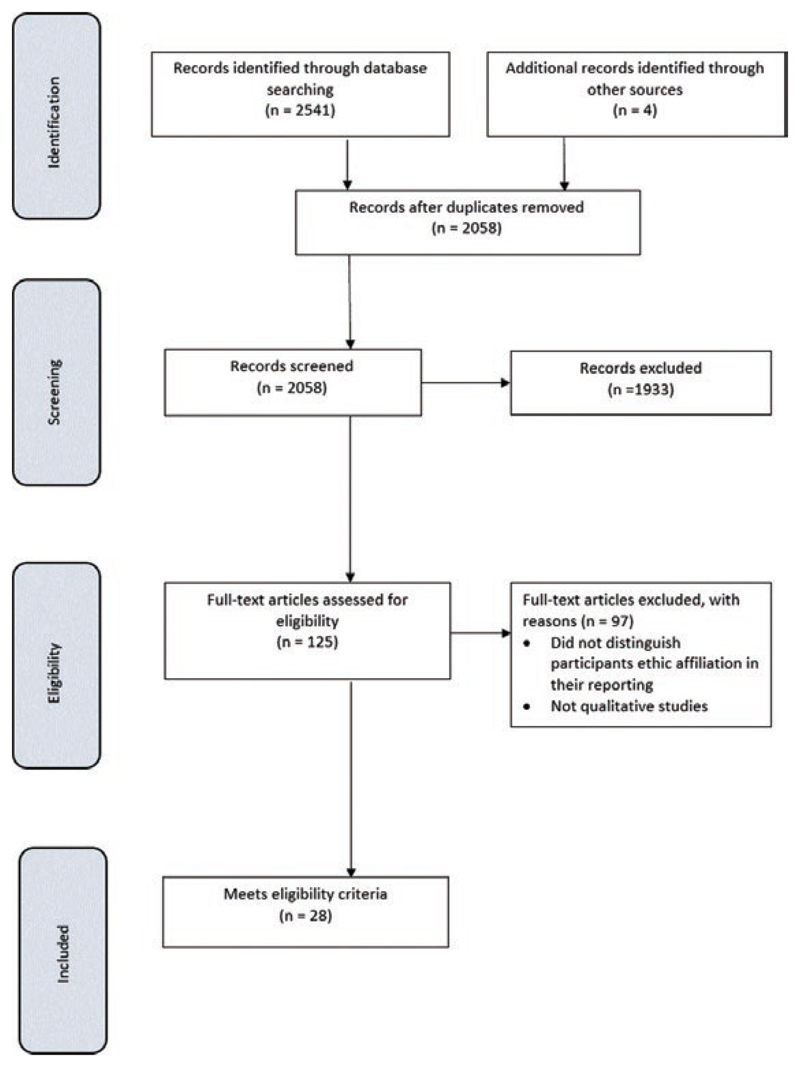
PRISMA diagram of study selection.

Figure 1 describes our search results. Table 1 shows the quality scores for each paper, which ranged between 3.5 and 6, with only four papers scoring full marks.

We included 28 papers about 25 studies conducted in United Kingdom (*n* = 7), United States (*n* = 17), and the Netherlands (*n* = 1). Study characteristics are described in Table 2. Seventeen papers were specific to people of BAC background with the majority from United States (*n* = 16) and one from United Kingdom. The majority of papers involved carers (*n* = 24), while only six articles included people with dementia and six involved members of the public. Three papers used a combination of observational with focus group methodology (Belgrave, Allen-Kelsey, Smith, & Flores, 2004) or interviews (Gerdner, Tripp-Reimer, & Simpson, 2007; Lindauer, Harvath, Berry, & Wros, 2016). The remaining papers used individual qualitative interviews or focus groups.

#### Ethnicity of participants

Only four papers provided a definition for the ethnic groups included (Adamson & Donovan, 2005; Fox, Hinton, & Levkoff, 1999; van Wezel et al., 2016, 2018). In total, the studies included elicited views from 492 people described as being of BAC ethnicity. Most (*n* = 350; 71%) were described as African American, in U.S.-based studies. In U.K. studies, participants were described as Caribbean (*n* = 45), as African/Caribbean (*n* = 44), African (*n* = 28), Black British (*n* = 7), or Indo-Caribbean (*n* = 1); and in the study from the Netherlands, participants were described as Surinamese Creole (*n* = 17). It was not possible to ascertain the number of participants of BAC ethnicity in two studies (Belgrave et al., 2004; Cheston et al., 2017). In the United Kingdom, where the origins of people of BAC ethnicity vary, only two studies reported participants’ background information (Berwald, Roche, Adelman, Mukadam, & Livingston, 2016; Lawrence, Samsi, Banerjee, Morgan, & Murray, 2011), with three not distinguishing African from Caribbean (Adamson, 2001; Adamson & Donovan, 2005; Mukadam, Cooper, Basit, & Livingston, 2011). In United States, most African Americans are African descents, but from various part of Africa and the World (Tishkoff et al., 2009); background details of participants were not provided in any of the studies.

#### Ethnicity of researchers

Arrangements to involve recruiters and/or interviewers with similar backgrounds as participants were reported in 6/25 (24%) of studies (Belgrave et al., 2004; Gerdner & Simpson, 2009; Gerdner et al., 2007; Lampley-Dallas, Mold, & Flori, 2001; Lindauer et al., 2016; van Wezel et al., 2016, 2018; Vickrey et al., 2007). We obtained confirmation of authors’ ethnicities for 12/28 (43%) of papers. Our analysis of the authors listed on the papers included, showed that 11/25 (44%) of studies involved at least one author who self-identified or was considered likely to be of Black ethnicity (Belgrave et al., 2004; Berwald et al., 2016; Cheston et al., 2017; Cloutterbuck & Mahoney, 2003; Epps, Rose, & Ruth, 2019; Fox et al., 1999; Gerdner & Simpson, 2009; Gerdner et al., 2007; Hughes, Tyler, Danner, & Carter, 2009; Lampley-Dallas et al., 2001; Moss et al., 2018; Stansbury, Harley, & Brown-Hughes, 2010; Stansbury, Marshall, Harley, & Nelson, 2010). Most of these (8/11) had a Black first or last author. Just over a quarter (3/11) considered the influence that their own ethnicity might have played in the formulation of the research outcome and reporting (Belgrave et al., 2004; Gerdner & Simpson, 2009; Moss et al., 2018). In total, just over half (14/25; 56%) of studies appeared to involve a Black person in the study procedures and analysis. While we accept that making racial and ethnic determination for those who did not reply to our request or who did not reveal this information in their reporting could be regarded as problematic, this was necessary in order to highlight that current research knowledge of BAC perceptions and experience of dementia is largely approached and reported from an outsider’s perspective.

### Themes

We identified four key themes that responded to our research question: “How do BAC adults with dementia or cognitive impairment, their families and other stakeholders perceive, understand, and experience dementia and dementia care services?”

These were: 1. *Dementia does not relate to me*, 2. *Inappropriate and disrespectful services*, 3. *Kinship and responsibility*, and 4. *Importance of religion*. All these themes were consistently present in papers conducted in the United Kingdom and United States where the majority of studies were conducted. Themes 2 and 4 were present in the Netherlands where only one study was conducted.

#### Dementia does not relate to me

Some people believed that dementia did not affect “Black people,” as they are not “seen” in the media and other outlets as a population at risk of developing the condition, or that it did not apply to their family:

When you talk about dementia… this is a White, old White people’s disease, it’s not seen as Black people have dementia. [BC man-UK] (Berwald et al., 2016)

I did not know, I was just talking about this with my brother before mama, I don’t think I had heard about it at all . . . ahh . . . I didn’t ask questions or nothing because it was not something that applied to me and my family, I did not really get into it before mama got it. [AA woman carer-USA] (Hughes et al., 2009)

Despite having heard of dementia people did not view it as a risk for their racial and ethnic group. This may indicate that people ascribe racial and ethnic attributes to illnesses based on how they are represented and whether they are encountered in their surroundings.

#### Inappropriate and disrespectful services

Services were viewed as constructed by and for White people, and even events that sought to be inclusive were often experienced as tokenistic occasions where ethnicity was objectified.

I spoke to someone because I said, “I don’t think Mummy’s whole needs are being met, with regards the cultural….” She’s happy, don’t get me wrong. And a few months ago, they did a Jamaican Day for her, but it was a Jamaican Day that involved all White people. Don’t get me wrong; they did their best; it was a great gesture. I appreciated it … but it would be great if someone within the system, the service … that I could find someone of West Indian background who could just sit with Mummy, talk with Mummy, be there with her. [BC woman carer-UK] (Cheston et al., 2017)

…they didn’t help me at all when it came to keeping over his things. That was a constant fight the whole time I was there … that was the most devastating thing … I shaved and cut his hair for awhile then they finally started shaving him. . . Those were important to me. It might not have meant anything to them, but I would tell them, “This is all I have. This is my husband. This is my baby. This is my all, and I want you to treat him as such. Treat him like a human being. Don’t throw him off in his room and fail to come see about him … Don’t scold him, don’t ever scold him. And just treat him like a human being even though he can’t relate to you.” [AA woman carer-USA] (Lampley-Dallas et al., 2001)

Dissatisfaction with health and social care services was high and reports of positive experiences were rare. Staff members were often perceived as unhelpful, unprofessional, and rude.

Welfare workers are the rudest people in the world. …People who work for the State are just rude. …They had me characterized when I went in there….I would never want to be involved with [welfare department] again. I refuse to go back there for help ever again. [AA man carer-USA] (Fox et al., 1999)

We got this negative attitude from the doctor that led me to taking him out of that facility and looking for another one. And then there was another one and we ended up in another. [AA unclear-USA] (Cloutterbuck & Mahoney, 2003)

But sometimes you go to the doctor; they don’t even have time for you. As you go in they write something, you haven’t got time. [BC woman-UK] (Berwald et al., 2016)

#### Kinship and responsibility

Where formal services fell short, needs for support were often met by a high reliance on kin, including family, friend, community, and churches. Caring for a relative was valued as a cultural trait and viewed as an act of love, an altruistic expression of respect and reciprocation for earlier care received:

…but the way we were brought up is that you look after your own sort of thing. We’ve always done that, we never sort of think about it cause you think to yourself, “gosh what are people going to think, that I don’t care about her” you just do it automatically cause it’s part of our culture, more or less you know, you don’t think of it any other way really.” […]…. “You’ve got to remind yourself, well you know, it’s your mother, she gave up an awful lot for you, you know, it’s small enough to do for her I don’t think you can ever repay your mother for what she’s done, I don’t think you can—but you can go some way towards showing her that you do care about her and you do appreciate her and all the things she has done I do it for my mother, out of sheer, because I love her and care for, care about her, not because of totally what she did. [BA/BC woman carer-UK] (Adamson & Donovan, 2005)

Look, where Surinamese people come from, the elderly are part of the family and stay part of it until they die. And whatever happens to them, whatever mental or physical condition they end up in later, the family should deal with it. Because they did the same for you when they were fit and strong. It’s a kind of repayment. [Surinamese Creole woman carer-NL] (van Wezel et al., 2016)

Well, I do it out of love. And the reward is that I do have her, even though she has this (disease). I wake up to her (in the house). [AA carer-USA] (Sterritt & Pokorny, 1998)

Even when this reliance on kin was onerous or costly, it transcended other obligations:

…I could never put my mother in a nursing home. I would just have to quit working before I’d put her in a nursing home. [AA carer-USA] (Gerdner & Simpson, 2009)

I’m there for her at any time at any, you know, which is really important, it’s not that you’re coming in and out, and the time she needs someone, there won’t be anyone there, so I think I’m doing an excellent job for her …some people would like to be independent to do some . . . try to do some things but you never know what they can do that will hurt them, really, so I think that when you are there throughout, it really helps. [BC woman carer-UK] (Lawrence, Murray, Samsi, & Banerjee, 2008)

There are moments when the care does weigh heavily on me. The moments when I see she is suffering, in particular. Those are tough. Not tough in the sense of physically tiring or whatever, not at all. It’s her suffering that weighs most heavily. [Surinamese Creole woman carer-NL] (van Wezel et al., 2016)

My brother sold his home … and he moved here. So everybody’s trying to help. [AA carer-USA] (Lampley-Dallas et al., 2001)

Carers were willing to go through great lengths and endure considerable hardships to care for an older relative. It appeared that this was done out of love and respect and was not experienced as being burdensome in itself. although it was challenging for respondents to see their relative suffering, it was unclear from our data whether this level of caring was expected by the older relative receiving the care.

#### Importance of religion

Spirituality and faith were often part of the context in which people understood and experienced dementia, and a source of healing and strength:

Well I think it’s a duty, because I think the lord have given me that problem, because he sends us here and we have to do a job. [BA/BC woman carer-UK] (Adamson & Donovan, 2005)

Every morning when I wake up, I pray and I hope that she’s still there. I get a lot out of it. [Surinamese Creole woman carer-NL] (van Wezel et al., 2016)

I believe that you can be healed through faith, but you must have that faith yourself. I don’t think nobody else can pray and heal you unless you have faith. I believe you have to have that faith before the illness [cognitive impairment] comes on you because if you can’t remember, you can’t ask God for forgiveness and to give you faith. [AA carer-USA] (Gerdner et al., 2007)

“I just put my trust in the good Lord, and He’ll give you strength,” and “When I’m tired and frustrated, I pray to God to give me the strength to deal with her.” [AA carer-USA] (Sterritt & Pokorny, 1998)

We found that there were many references to religion and to god. This finding, however, might have been influenced by the researchers’ choice of a religion or spirituality research topic given that a number of the studies specifically explored or addressed religion.

## Discussion

The focus of this review was to foreground nuance and “cultural” distinctiveness as well as the typicality of how BAC adults understand and experience dementia and dementia care services. To our knowledge, this is the first systematic review analyzing this topic and which takes the racial and ethnic context in which studies were conduction into consideration. We found that dementia in relation to ethnicity is largely researched within a context of ethnic discordance between participants and investigators. The terms used to describe the ethnic and cultural grouping of study populations vary across locations, which made synthesizing and interpreting study findings difficult. In light of these difficulties, we identified four key themes that carried cultural overtones and characterized how the people studies described as “Black” understood and experienced dementia.

It emerged from the data that the risk of developing dementia went unacknowledged because some people viewed it as an “old White people’s disease [as] it is not seen as Black people have dementia” (Berwald et al., 2016; Cheston et al., 2017). Such belief coheres with the lack of racial and ethnic diversity in public health resources and the media in the representation of dementia, which contrasts greatly with other chronic condition such as HIV where portrayal of African Americans prevails over other ethnic groups (Clarke, McLellan, & Hoffman-Goetz, 2006; Stevens & Hull, 2013). Generally, low dementia literacy is correlated with ethnicity and reported to be prevalent in BAC populations and proposed as an explanation for beliefs that depart from the medical model of dementia (Ayalon & Areán, 2004; Mukadam et al., 2011; Mukadam, Waugh, Cooper, & Livingston, 2015; Werner, Goldstein, Karpas, Chan, & Lai, 2014; Werner, Mittelman, Goldstein, & Heinik, 2012). However, we did not find evidence of unusually low levels of dementia understanding in this review that had not been reported in other ethnic groups in studies conducted in France (Breining et al., 2014), Istanbul (Şahin et al., 2006), Ireland (McParland, Devine, Innes, & Gayle, 2012), and Australia (Bettens, Ownsworth, Hohaus, & McKendry, 2014). In fact, most participants included in this review had heard of dementia before being involved in a diagnosis process. Thus, ethnic affiliation does not appear to be a reliable factor in determining understanding of dementia.

We report consistently negative evaluation and experience of health and social care services, which contrasted to positive experiences of the care received from or provided by the immediate social network. Bad experiences, service provision inequalities, anticipated discrimination, structural disadvantages, mistrust, and poor patient–provider relationship all contributed to delays and discouragements in accessing resources. Formal support was often viewed as failing to provide adequate services to BAC families, even when tokenistic efforts attempted to conceal the lack of diversity and cultural adaptation of services. Previous studies have reported adverse experiences with health and social care services in minority ethnic groups as barriers to helpseeking for dementia (Berwald et al., 2016; Cloutterbuck & Mahoney, 2003; Hinton, Franz, & Friend, 2004). Acknowledging the “invisibility” of Black older people in dementia and social care services, others have called for policies prioritizing the development of appropriate services to meet the needs of this population (Moriarty, Sharif, & Robinson, 2011). To be effective, these policies will need to recognize that people of BAC ethnicity share strong family values and norms about the responsibility of the family to care, respect, and protect the older adults (Werner et al., 2014). Because family/close social network influence can act both as a facilitator and a deterrent to accessing services (Werner et al., 2014), service providers should look toward fostering collaboration with family members and building relationship with communities.

We also found that religiosity was important in understanding and managing dementia as people drew strength and support in prayer and religion. There were expectations of positive outcome, relief an even healing from the burden of dementia in the association with God and their churches. Previous studies have shown similar strong endorsement of religious coping patterns and engagement with religion in dealing with memory problems in African Americans (Chatters, Taylor, Jackson, & Lincoln, 2008; Sullivan & Beard, 2014).

The reliance on kin for care, support, and respite was also an important aspect of managing dementia. BAC carers experience less emotional burden from caring for relative with dementia than their White counterparts, despite sharing similar physical health effects of caregiving (Covinsky et al., 2003; Roth, Haley, Owen, Clay, & Goode, 2001; Siegler, Brummett, Williams, Haney, & Dilworth-Anderson, 2010). They tend to find more satisfaction in their caregiver role than White caregivers (Sörensen & Pinquart, 2005). Work on anticipatory grief suggests that African American carers retain a strong emotional attachment to their relative right through the trajectory of dementia, whereas their White counterparts detach emotionally before their relative with dementia die (Ross & Dagley, 2009).

Taken together, these findings suggest that cultural characteristics may be a function of the differential evaluation of the caregiver role and religious endorsement, which likely can raise the threshold for coping with the burden of dementia. Understanding the importance of religiosity and family in BAC families may be critical in engaging with this group. In the United Kingdom, Black older adults are still invisible in dementia services despite multiple campaigns and advocacy activities (Moriarty et al., 2011; Truswell, 2013), which indicates that interventional procedures need to better target their efforts if they are to meet the needs of BAC older adults with dementia and their families.

## Implications

If research and practice are to create truly effective culturally tailored interventions and services for minority ethnic populations, they must be able to describe and distinguish the ethnicity and cultural backgrounds of these populations. Until now, studies have tended to homogenize minority populations in their analyses and reporting which renders interpreting their findings challenging and often blurring cultural nuances. For example, in United Kingdom, the BME classification includes people who have various origins and cultures, such as African from different parts of the African continent and Caribbean from multiple islands. The BME label also includes South Asians (from Pakistan, India, Sri Lanka, and Bangladesh), White other than White British, and other minority populations. Classification devices and labels are useful in research as in practice, as they can help understand commonalities and differences and provide some indications of broad characteristics of the populations being discussed. However, they should not vitiate the need for background details and additional information about people as these are often more relevant and descriptive of individuals than predefined categories and labels. Both research and practice must exercise caution when considering study findings as they might not apply to all individuals included in a given group.

The large proportion of included studies was conducted by researchers who had little in common with participants. Just over half (56%) of studies investigating experiences of dementia of adults from BAC ethnicity appeared to consult or involve a person who self-identified as coming from a BAC background during the study procedures or analysis. There is much debate about the insider–outsider perspectives in research, particularly in research into race and ethnicity, and the need for a recognition of the advantages and disadvantages of occupying either position, including the notion of the space between that challenges the dichotomy of insider versus outsider status (Beoku-Betts, 1994; Collins, 2002; Dwyer & Buckle, 2009; Flores, 2018; Serrant-Green, 2002). In conducting qualitative research it is essential to create and maintain a context in which participants feel comfortable enough to share freely with researchers and ensure actual or anticipated obstacles are kept to a minimum, whether it be about communication, language, cultural competence, and more (Bonner & Tolhurst, 2002). This matters because matching racial makeup between participant and investigator has positive effects on accuracy and relevance of findings as relational intimacy and understanding of group culture facilitate communication and comprehension of meanings and nuances (Bonner & Tolhurst, 2002; Botsford, 2015). Researchers should consider when investigating ethnicity in relation to dementia that participants often feel more comfortable and willing to speak to a researcher who share similar backgrounds as them. Clearly, there is much strength in fostering commonality and shared values between researcher and participant to gain easier access to richer quality data, which is an essential aspect in qualitative research.

Much of research about Black and other minority ethnic groups is conducted within a context that makes assumptions about racial and ethnic identities and categories. Furthermore, these assumptions are extended to attributes, characteristics, behaviors, and practices that are found in studies in this field. Such framing can implicitly or even explicitly determine the direction of research as well as have an impact on the presentation and interpretation of findings. This may occur even when other more important factors may be at play. Therefore, we would suggest that researchers need to be aware of the contextual nature of the categories that they are using in this field of study if their findings are going to be culturally, racially, or ethnically meaningful.

Dementia in relation to ethnicity has largely been studied in the context of help-seeking and within a biomedical model which have firmly situated people of BAC ethnicity in an unfavorable position. The majority of studies we included were conducted by research team members whose racial and ethnic profiles did not match those of the participants. In the light of the stark underrepresentation of BAC people in the research community, this is very telling that even most research about BAC experiences does not involve researchers from BAC background. Themes of dementia diagnosis and services feeling less relevant to participants than the majority population resonated across studies. We caution against racializing these findings, which may apply to many minority groups.

## Figures and Tables

**Table 1:** Quality Assessment of Included Studies Using the Critical Appraisal Skills Programme—Qualitative Checklist—Section A—Validity

	Quality control						
Papers	1	2	3	4^a^	5^a^	6^a^	Total score
Adamson (2001)	1	1	1	0.5	0.5	0	4
Adamson & Donovan (2005)	1	1	1	0.5	0.5	0	4
Lawrence et al. (2008)	1	1	1	0.5	0.5	0	4
Lawrence et al. (2011)	1	1	1	1	0.5	0	4.5
Mukadam et al. (2011)	1	1	1	0.5	0.5	0	4
Berwald et al. (2016)	1	1	1	1	1	0	5
Cheston et al. (2017)	1	1	1	0.5	0.5	0	4
Sterritt & Pokorny (1998)	1	1	1	0	0.5	0	3.5
Fox et al. (1999)	1	1	1	0.5	0.5	0	4
Lampley-Dallas et al. (2001)	1	1	1	0.5	1	0.5	5
Cloutterbuck & Mahoney (2003)	1	1	1	0.5	0.5	0	4
Belgrave et al. (2004)	1	1	1	1	1	1	6
Toth-Cohen (2004)	1	1	1	0.5	0.5	0	4
Jett (2006)	1	1	1	0.5	0	0	3.5
Gerdner et al. (2007)	1	1	1	0.5	0.5	0.5	4.5
Vickrey et al. (2007)	1	1	1	0.5	1	0.5	5
Gerdner & Simpson (2009)	1	1	1	1	1	1	6
Hughes et al. (2009)	1	1	1	0.5	0.5	0	4
Stansbury, Harley, et al. (2010)	1	1	1	1	0.5	0	4.5
Stansbury, Marshall, et al. (2010)	1	1	1	1	0.5	0	4.5
Sullivan & Beard (2014)	1	1	1	0.5	0.5	0	4
Roberts et al. (2015)	1	1	1	0.5	0.5	0	4
Lindauer et al. (2016)	1	1	1	1	1	1	6
Potter et al. (2017)	1	1	1	0.5	0.5	0	4
Moss et al. (2018)	1	1	1	1	1	1	6
Epps et al. (2019)	1	1	1	0.5	0.5	0	4
van Wezel et al. (2016)	1	1	1	1	1	0.5	5.5
van Wezel et al. (2018)	1	1	1	1	1	0.5	5.5

a *Note:* Partial point was given if studies did not match recruiter–participant ethnicity, for example, by making use of Black informant/recruiter/interviewer in addition to standard methodology or failed to address the potential influence on findings of the ethnic discordance between researcher and participant.

**Table 2:** Data Extracted From 28 Papers Included in the Review

Author & location	Aims	Total participants (any ethnicity)	Total Black participants and characteristics
Adamson (2001); United Kingdom	To explore awareness, recognition, and understanding of dementia	30 BAC, SA	18 carers 15F; 3M
Adamson & Donovan (2005); United Kingdom	To explore experiences of informal caring for older relative with dementia	36 BAC, SA	21 carers ^(b)^
Lawrence et al. (2008); United Kingdom	To explore attitudes, experiences, and needs of carers of people with dementia	32 BC, SA, WB	10 carers 9F; 1M
Lawrence et al. (2011); United Kingdom	To explore attitudes, experiences, and beliefs of dementia	30 PwD BC, SA, WB	11 PwD 8F; 3M
Mukadam et al. (2011); United Kingdom	To explore beliefs and attitudes to help-seeking for dementia	18 BA/BC, SA, W, Ao, Chi	5 carers ^(b)^
Berwald et al. (2016); United Kingdom^a^	To identify barriers to help-seeking for memory problems	50 BA, BC, BB, 1IndoC	42 members of the public 4 PwD 4 carers 30F; 20M
Cheston et al. (2017); United Kingdom	To establish dementia experiences and needs of people BME	48 ^(b)^ BC, SA, Chi	2 PwD 8 carers ^(b)^ members of the public
Sterritt & Pokorny (1998); United States^a^	To explore meaning of caregiving to AA caregivers of relatives with Alzheimer’s disease	9 AA	9 carers 9F
Fox et al. (1999); United States^a^	How race and ethnicity matter in recognition, meaning, and responses to dementia in AA caregivers?	10 AA	10 carers ^(b)^
Lampley-Dallas et al. (2001); United States^a^	To assess perceived needs of AA caregivers of people with dementia and expectations of health care system	13 AA	13 carers 11F; 2M
Cloutterbuck & Mahoney (2003); United States^a^	To explore perceptions and experiences of AA caregivers in getting dementia diagnosis for relatives	7 AA	7 carers 5F; 2M
Belgrave et al. (2004); United States^a^	Explanations of Alzheimer’s disease from perspectives of AA carers and patients	36 families AA	41 carers ^(b)^ PwD
Toth-Cohen (2004); United States^a^	To explore factors that may influence appraisal of upset in Black caregivers of people with dementia in response to relatives’ memory and behavior problems	15 AA	15 carers 12F; 3M
Jett (2006); United States^a^	To discover the cultural and linguistic variations in the definition, recognition, explanation of, and response to, dementia as it is experienced in the AA community	14 AA	14 members of the public 13F; 1M
Gerdner et al. (2007); United States^a^	To describe experience of AA caregiver understanding of chronic confusion and experience of family caregiving	15 carers AA	15 carers 11F; 4M
Vickrey et al. (2007); United States	To explore contrasts and commonalities in caregiving experiences across four racial/ethnic groups, and to obtain data to aid in designing future interventions to improve the quality of dementia care	47 carers AA, W, His, Chi	19 carers 34F; 13M
Gerdner & Simpson (2009); United States^a^	To explore the primary concerns related the elder’s condition and access to and use of health and community services	15 AA	15 carers 11F; 4M
Hughes et al. (2009); United States^a^	To explore factors associated with decision to seek a diagnosis for a family member with dementia in AA caregivers	17 AA	17 carers 14F; 3M
Stansbury, Harley, et al. (2010); United States^a^	To determine AA clergy’s awareness of Alzheimer’s disease and willingness to provide support to elders and their family/caregivers	9 clergy (Baptist) AA	9 members of the public 9M
Stansbury, Marshall, et al. (2010); United States^a^	To explore rural AA clergy knowledge and beliefs of Alzheimer’s disease	9 clergy (Baptist) AA	9 members of the public 9M
Sullivan & Beard (2014); United States	To examine how sociocultural aspects of religion/spirituality influence experiences of living with Alzheimer’s disease for diverse diagnosed seniors and their families	75 AA, W	43 PwD & carers 42F; 1M
Roberts et al. (2015); United States	To explore how three ethnoracial communities experience cognitive decline and aging	75 people AA, W, Lat	16 carers & members of public ^(b)^
Lindauer et al. (2016); United States^a^	To explore the meaning AA caregivers ascribe to the dementia-related changes in their care recipients	11 AA	11 carers ^(b)^
Potter et al. (2017); United States^a^	How AA families recognize and respond to perceived changes in their older relatives’ cognitive abilities?	27 families (67 people) AA	27 PwD 18F; 9M 40 carers 34F; 6M
Moss et al. (2018); United States^a^	To examine understanding of end-of-life decision-making terminology among family caregivers of AA older adults with dementia	18 AA	18 carers 17F; 1M
Epps et al. (2019); United States^a^	To examine and characterize family networks of AA family caregivers who provide care to family members with dementia	18 families AA	26 carers ^(b)^
van Wezel et al. (2016); The Netherlands	To describe the perspectives of female family carers of three ethnic groups about providing care to a relative with dementia	69 Surinamese Turkish Moroccan	17 carers 17F
van Wezel et al. (2018); The Netherlands	To explore how female family carers from three ethnic groups explain and describe the dementia of their close relative	69 Surinamese Turkish Moroccan	17 carers 17F

*Note:* AA = African American; BA = Black African; BC = Black Caribbean; BB = Black British; AC = African and Caribbean; BAC = Black African and Caribbean; SA = South Asian; W = White; WB = White British; Chi = Chinese; Ao = Asian other; Lat = Latino; PwD = people with dementia; BME = Black and Minority Ethnic.

a Papers specific to Black participants.

(b) Number or details not specified in the study.
